# Gram-GAN: Image Super-Resolution Based on Gram Matrix and Discriminator Perceptual Loss

**DOI:** 10.3390/s23042098

**Published:** 2023-02-13

**Authors:** Jie Song, Huawei Yi, Wenqian Xu, Bo Li, Xiaohui Li

**Affiliations:** School of Electronics and Information Engineering, Liaoning University of Technology, Jinzhou 121001, China

**Keywords:** super-resolution, generative adversarial network, perceptual loss

## Abstract

The solution of a high-resolution (HR) image corresponding to a low-resolution (LR) image is not unique in most cases. However, single-LR–single-HR supervision is widely adopted in single-image super-resolution (SISR) tasks, which leads to inflexible inference logic of the model and poor generalization ability. To improve the flexibility of model inference, we constructed a novel form of supervision, except for the ground truth (GT). Specifically, considering the structural properties of natural images, we propose using extra supervision to focus on the textural similarity of the images. As textural similarity does not account for the position information of images, a Gram matrix was constructed to break the limitations of spatial position and focus on the textural information. Besides the use of traditional perceptual loss, we propose a discriminator perceptual loss based on the two-network architecture of generative adversarial networks (GAN). The difference between the discriminator features used in this loss and the traditional visual geometry group (VGG) features is that the discriminator features can describe the relevant information from the perspective of super-resolution. Quantitative and qualitative experiments were performed to demonstrate the effectiveness of the proposed method.

## 1. Introduction

Single-image super-resolution reconstruction (SISR) is a classic image processing task. Its target is to obtain a corresponding high-resolution (HR) image through some logical inference based on an existing low-resolution image (LR). In the current information age, people have increasingly higher requirements for image resolution (e.g., medical, monitoring and multimedia industries), which makes SISR have high practical value.

In recent years, with the rapid development of deep learning, neural network models related to SISR have emerged in an endless stream. The pioneering work was a proposal of SRCNN [1], which first applied convolutional neural networks to super-resolution (SR) tasks and substantially improved the quality of reconstructed images. Thereafter, a large number of PSNR-oriented methods [2,3,4,5,6,7] have emerged, which have the uniform property of a loss function consisting of a single mean square error (MSE), pixel-wise loss [8,9]. Although neural networks have strong learning ability, the models so far have focused only on maximizing PSNR, resulting in the problem of over-smooth images.

Perception-driven methods [10,11,12,13,14,15] are proposed to solve the over-smoothness problem. These methods extract image features through the first half part of the pre-trained partial visual geometry group (VGG) network [16], and then construct the perceptual loss. The loss makes the network have the ability to reason about high-frequency texture details compared with pixel-wise loss, so as to obtain visual effects more in line with human perceptual habits.

Although perception-driven methods substantially improve the visual quality of images, the use of one-to-one supervision in most models is not reasonable. For one thing, LR images are not in a fixed one-to-one relationship with HR images. For another, multiple HR images may be downsampled to the same LR image (the downsampling method is uncertain). Therefore, one-to-many supervision needs to be constructed to improve the flexibility of model inference.

To solve the above problem, Li et al. [17] implemented one-to-many supervision based on the similarity between patches. However, its similarity measurement standard was the Euclidean distance (i.e., treating all content information within patches equally), which led to the possibility that additional selected supervised patches may differ from the ground-truth (GT) patch in details (cf. the image over-smoothness problem in PSNR-oriented methods), thus affecting the visual quality of the images. In this paper, to produce higher quality images, we use the Gram matrix to develop a supervision that emphasizes textural information. The model can flexibly generate more realistic textures and avoid some distorted structures under this supervision. In addition, we believe that the VGG features used in traditional perceptual loss are not fully adapted to SR models. The original purpose of these features was to be applied to the image recognition task [18,19], which makes the feature type required by the model in the SR task not rich enough. To enhance the richness of the feature types, we propose using the features of the middle layer of the discriminator [20] for the training of the generator. With the combined effect of the discriminator and VGG features, the network can learn richer inference logic, and thus generate more natural textural details.

In this paper, we refer to the models obtained by the above two proposed methods as Gram-GAN. Gram-GAN is compared with a large number of perception-driven methods to demonstrate its advancement, and ablation experiments are conducted to verify the necessity of each method.

The main contributions of this paper are itemized as follows:

1. In order to improve the flexibility of model inference, this paper proposes a method of constructing a Gram matrix for patches to formulate another supervision except for GT. This supervision ignores the position information of images and focuses only on texture information, which can reduce the generation of distorted structures with a large deviation from GT.
2. We propose a discriminator perceptual loss dedicated to the SR task based on the two-network architecture of generative adversarial networks (GAN), which can give the network some additional inference logic from the SR perspective compared with traditional perceptual loss.
3. Massive advanced perception-driven methods are used to compare their performance with Gram-GAN to demonstrate the advancement of the proposed method, and ablation experiments are performed to verify the respective necessity of the constructed extra supervision and discriminator perceptual loss.

## 2. Related Work

In this section, we introduce the current SR methods from two perspectives, which are PSNR-oriented methods [2,3,4,5,6,7] and perception-driven methods [10,11,12,13,14,15,17].

### 2.1. PSNR-Oriented Methods

With the proposal of SRCNN [1], deep learning in SR tasks have become increasingly mature, and massive models aimed at improving PSNR values have been proposed. In particular, Kim et al. [2] proposed VDSR, which improved the performance of the model by significantly increasing the number of network layers. Ledig et al. [3] combined the ideas of ResNet [21] and proposed the SRResNet. Zhang et al. [4] proposed RCAN, which constructed a channel attention module to focus on improving the PSNR value. Hu et al. [5] proposed Meta-SR to achieve the effect of upsampling images to arbitrary sizes. Li et al. [6] proposed a feedback framework to gradually refine the super-resolved results.

### 2.2. Perception-Driven Methods

It has been found that most PSNR-oriented methods suffer from a severe image over-smoothness problem, which is inextricably linked to the using a single pixel-wise loss. To enable the model to have the ability to reason about texture details, perceptual loss [10] was proposed. The idea was to use a pre-trained VGG model to extract image features and then compare the similarity of deep features between the predicted image and the GT image. With the great success of perceptual loss in SR, a series of perception-driven approaches have emerged. Ledig et al. [3] proposed SRGAN, which applied both GAN [20] and perceptual loss to the SR task to further improve the visual quality of images. Wang et al. [11] made improvements based on SRGAN and proposed ESRGAN. In particular, the modification of the network structure substantially improved the learning ability of the model, and thus reconstructed the finer textures. Rad et al. [12] made adjustments to the composition of perceptual loss and proposed a target perceptual loss based on object, background and boundary labels. Importantly, Li et al. [17] considered that one-to-one supervision was not the most reasonable way, and proposed the Beby-GAN with one-to-many supervision. However, the extra supervision of the method was selected by finding the patches that had the shortest Euclidean distance from the estimated patches. This easily generated texture details that differed significantly from GT patches. In addition, VGG features were oriented towards image recognition tasks, so the current perceptual loss did not enable the model to reason about other details in the images. The construction of additional types of perceptual loss is crucial to enhance inference capability of the model. To this end, we propose using Gram-GAN to solve these problems.

## 3. Methods

**Figure 1:**
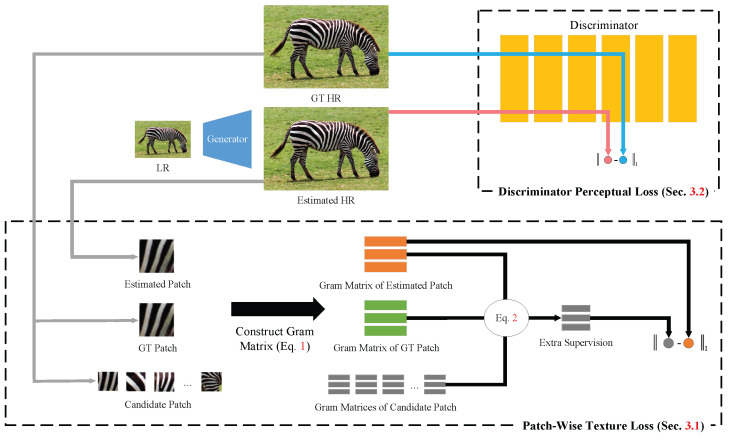
The whole framework of Gram-GAN. The discriminator perceptual loss is constructed by the output of the middle layer of the discriminator. The patch-wise texture loss gives texture-based supervision to the estimated patch.

The whole framework of the proposed Gram-GAN is constructed based on GAN, as shown in Figure 1. The overall network consists of a generator and discriminator. The generator uses the RRDB [11], with a strong learning capability to adapt a series of complex loss functions, and the discriminator uses a variant of the VGG network. In this section, we first introduce extra supervision and construct the patch-wise texture loss. Then, we illustrate a novel discriminator perceptual loss. Finally, the other loss functions used in the model are mentioned.

### 3.1. Extra Supervision Based on Gram Matrix

In order to enhance the flexibility of model inference through one-to-many supervision, a practical extra supervision needs to be added on top of the original GT image supervision set on the estimated images. Inspired by [17], we set this extra supervision from the patch. To fit the SR task, we considered that texture similarity needed to be given more attention, rather than all content information being treated equally when finding extra supervision. The reason for this was that in most natural images, due to the limitation of location information, it is much harder to find a patch similar to the estimated patch in content than texture, except for the GT patch. Therefore, to construct another kind of supervision more reasonably, this paper proposes to construct the corresponding Gram matrix for each patch to achieve the purpose of ignoring the location information and focusing only on texture information, as follows.

First, the patch is defined as $p\in R^{S\times C}$, where *S* represents the dimension after the multiplication of height and width (the two dimensions are combined) and *C* represents the number of channels. The Gram matrix construction function can be expressed as

(1) $$G\left(p\right)=p^{T}p.$$

The Gram matrix used in this paper was not constructed based on the feature extraction mechanism of the pre-trained network, but was directly constructed from the original features. Considering that every patch carries a small amount of information, the Gram matrix constructed by the original features was sufficient to distinguish between different texture properties. Therefore, the use of a complex feature extraction mechanism was unnecessary.

Then, the selection method for the extra supervision was formulated by joint decision of the GT patch in [17] and the estimated patch. However, the difference is that the measure of similarity between patches is no longer based on the full content, but on the texture. The extra supervision in the i-th iteration can be represented as

(2) $$p_{i}^{*}=\underset{p\in O}{\arg\min}[\alpha {∥G\left(p\right)-G\left(g_{i}\right)∥}_{2}^{2}+\beta {∥G\left(p\right)-G\left(e_{i}\right)∥}_{2}^{2}],$$

where $g_{i}$ and $e_{i}$ represent the GT patch and estimated patch in the i-th iteration, respectively. α and β represent the corresponding weights and O denotes the candidate database. In particular, besides the patch set composed of the GT patch and downsampled GT patch, we added a patch set with affine transformation [22] to the candidate database to enrich the selectable texture types. The specific affine transformation operation can be formulated as

(3) $$\left[\begin{array}{c} x^{\left(1\right)}\\ y^{\left(1\right)}\\ 1 \end{array}\right]=\left(\lambda N+I\right)\left[\begin{array}{c} x^{\left(0\right)}\\ y^{\left(0\right)}\\ 1 \end{array}\right],$$

where $x^{\left(0\right)}$ and $y^{\left(0\right)}$ represent the original horizontal and vertical coordinates of the GT patch, respectively. $I\in R^{3\times 3}$ is the identity matrix. $x^{\left(1\right)}$ and $y^{\left(1\right)}$ represent the horizontal and vertical coordinates through affine transformation, respectively. $N\in R^{3\times 3}$ is a random matrix conforming to the standard normal distribution, and λ is used to control the magnitude of the affine transformation. Some unconventional distorted patches were added to the candidate dataset after this affine transformation, which could be considered as new texture types for selection.

**Figure 2:**
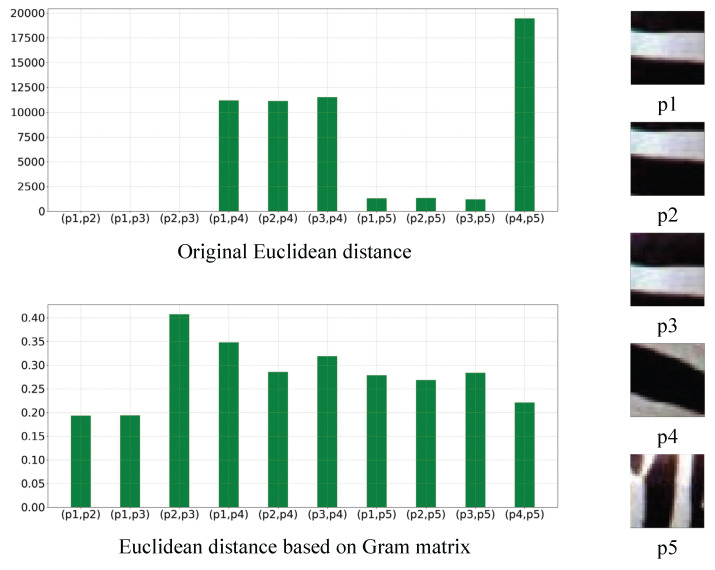
Comparison of the two distance calculation approaches with different texture information. (p1, p2) represents the distance between patch p1 and p2, and so on.

To highlight the benefits of pre-constructing the Gram matrix when selecting extra supervision, in Figure 2, we show the difference between two methods of calculating the Euclidean distance, which include the use of the Gram matrix and direct calculation. p1, p2 and p3 are patches containing the same type of textures, and p4 and p5 are patches containing other types of textures. When measuring the distance between two patches, we expect that the distance between patches with similar textures would be much smaller than the distance between patches with non-similar textures. From the figure, it can be observed that the direct calculation of Euclidean distance cannot distinguish the patches with similar and non-similar textures well, and the desired effect can be better achieved only after the Gram matrix is constructed. Therefore, the degree of similarity of the different textures can be measured more accurately after the introduction of the Gram matrix, which helps to select the extra supervision that can bring more benefits to the model.

Finally, for each patch pair $\left(e_{i},p_{i}^{*}\right)$ obtained by Equation (2), the corresponding patch-wise texture loss is represented as

(4) $$L_{PT}\left(e_{i},p_{i}^{*}\right)={∥G\left(e_{i}\right)-G\left(p_{i}^{*}\right)∥}_{1}.$$

### 3.2. Discriminator Perceptual Loss

The features extracted by the pre-trained VGG network were initially dedicated to the image recognition task, which made these features focus on the parts that were useful for this task. However, the SR task requires that the richer feature types predict every detail of the images. Therefore, the composition of the perceptual loss should not rely only on VGG features, but also on some additional features extracted for the SR task. Based on the above theory, this paper proposes to use the discriminator in GAN to construct a novel perceptual feature. Specifically, the discriminator in each iteration is used to extract features, and the discriminator perceptual loss corresponding to the i−th iteration can be represented as

(5) $$L_{DP}=\sum_{k}{∥D_{k}^{\left(i\right)}\left(x_{SR}\right)-D_{k}^{\left(i\right)}\left(x_{GT}\right)∥}_{1},$$

where $D_{k}^{\left(i\right)}$ represents the feature output by the k-th convolutional layer (after activation) of the discriminator at the i-th iteration. $x_{SR}$ and $x_{GT}$ represent the estimated and GT image, respectively.

**Figure 3:**
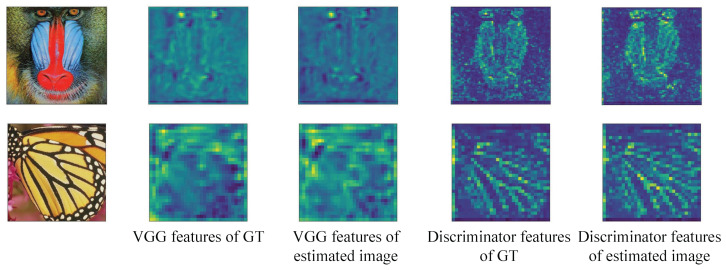
Comparison of VGG features and discriminator features on the estimated image and GT image. The estimated image is generated by RRDB.

Figure 3 shows the difference between VGG features and discriminator features, and the difference between the two features is very obvious. VGG features only highlight features useful for image recognition work (e.g., eyes), whereas the discriminator features emphasize features relevant to SR tasks (e.g., textures). Compared with VGG features, the discriminator features can highlight the differences between estimated images and GT images in more detail from different perspectives. Therefore, the network makes inferences based on more types of features after discriminator perceptual loss is added, to further improve the quality of the estimated images.

### 3.3. Other Loss Functions

#### 3.3.1. Perceptual Loss

In addition to the use of discriminator perceptual loss proposed in Section 3.2, traditional perceptual loss [10] is also considered in this paper, which is represented as

(6) $$L_{P}=\sum_{i}\lambda_{i}{∥\Phi_{i}\left(x_{SR}\right)-\Phi_{i}\left(x_{GT}\right)∥}_{1},$$

where $\Phi_{i}$ represents the i-th activation layer in the pre-trained VGG19 network. $\lambda_{i}$ represents the coefficient of balance loss. Following [17], the layers we considered included $conv_{3\_4}$, $conv_{4\_4}$ and $conv_{5\_4}$, and the corresponding scaling coefficients were $\frac{1}{8}$, $\frac{1}{4}$ and $\frac{1}{2}$, respectively.

#### 3.3.2. Adversarial Loss

For adversarial training under the GAN [20] mechanism, we used relativistic average GANs (RaGANs) with region-perceptual ability based on the ideas proposed in [17,22]. The loss functions of RaGANs can be represented as

(7) $$L_{D}=-E_{x_{r}^{\mathrm{mask}\;}∼P}\left[\log\left(D_{Ra}\left(x_{r}^{\mathrm{mask}\;}\right)\right)\right]-E_{x_{f}^{\mathrm{mask}\;}∼Q}\left[\log\left(1-D_{Ra}\left(x_{f}^{\mathrm{mask}\;}\right)\right)\right],$$

(8) $$L_{G}=-E_{x_{r}^{\mathrm{mask}\;}∼P}\left[\log\left(1-D_{Ra}\left(x_{r}^{\mathrm{mask}\;}\right)\right)\right]-E_{x_{f}^{\mathrm{mask}\;}∼Q}\left[\log\left(D_{Ra}\left(x_{f}^{\mathrm{mask}\;}\right)\right)\right],$$

where

(9) $$D_{Ra}=\left\{\begin{array}{cccc} \mathrm{Sigmoid}\left(C\left(x\right)-E_{x_{f}^{mask}∼Q}C\left(x_{f}^{\mathrm{mask}\;}\right)\right), & x & \;\mathrm{is}\; & \;\mathrm{real}\;\\ \mathrm{Sigmoid}\left(C\left(x\right)-E_{x_{r}^{\mathrm{mask}\;}∼P}C\left(x_{r}^{\mathrm{mask}\;}\right)\right), & x & \;\mathrm{is}\; & \;\mathrm{fake}\;, \end{array}\right.$$

where C(·) is the discriminator used to determine the true or false image, $x_{r}^{mask}$ represents real data that is sampled from distribution P and partially masked and $x_{f}^{mask}$ represents the fake data that is sampled from distribution Q and partially masked. The binary mask that masks the true and false data can be represented as

(10) $$M_{i,j}=\left\{\begin{array}{cc} 1, & \mathrm{std}\left(B_{i,j}\right)\geq \delta \\ 0, & \mathrm{std}\left(B_{i,j}\right)\leq \delta , \end{array}\right.$$

where $B_{i,j}$ represents the patch with coordinates (i,j) obtained by unfolding the image (length and width are fixed). δ is the predefined threshold, and std(·) is the operation of calculating the standard deviation. The value of δ and size of the patch were set to 0.005 and 11×11, respectively [17].

#### 3.3.3. Content Loss

The content loss was used to evaluate the *ℓ*1-norm distance between the estimated and GT image, and was formulated as

(11) $$L_{C}={∥x_{SR}-x_{GT}∥}_{1}.$$

The reason for using *ℓ*1-norm instead of *ℓ*2-norm is as follows: The advantage of the *ℓ*1-norm over the *ℓ*2-norm is that the *ℓ*1-norm is insensitive to outliers. As this paper uses a GAN-based network architecture, the model is trained in an adversarial way. This adversarial training inevitably results in some outliers. Therefore, we needed to use *ℓ*1-norm to reduce the impact of outliers as much as possible to enhance the stability of the model training.

#### 3.3.4. Overall Loss

Based on the above sections, the overall loss of the generator is

(12) $$L=\eta_{1}L_{PT}+\eta_{2}L_{DP}+\eta_{3}L_{P}+\eta_{4}L_{G}+\eta_{5}L_{C},$$

where $\eta_{1}=1.0$, $\eta_{2}=1.0$, $\eta_{3}$ = 1.0, $\eta_{4}=0.005$ and $\eta_{5}=1.0$. In particular, the reason why the weight of $L_{G}$ was taken as 0.005 is as follows: For the weights in Equation (12), the purpose was to ensure the consistency of magnitudes among the losses and to prevent the phenomenon that some losses do not bring gains to the model. Regarding the acquisition of specific values, our strategy was to take the value of the initial state of the loss as a benchmark and calculate the corresponding weight values with the goal of balancing the magnitude differences. As the value of $L_{G}$ in the initial state is larger compared with other losses, setting the weight to 0.005 can better balance the effect of each loss.

## 4. Experiments

### 4.1. Datasets and Similarity Measures

The training set was from 800 high-resolution images of the widely used dataset DIV2K [23]. All images were cropped by sliding window and expanded to obtain 44,226 non-overlapping sub-images with the size of 192×192. The test sets were Set5 [24], Set14 [25], BSD100 [26] and Urban100 [27], which had 5 images, 14 images, 100 images and 100 images, respectively.

In this paper, we used four evaluation metrics. The ones with reference objects were peak signal-to-noise ratio (PSNR), structure similarity (SSIM) [28], learned perceptual image patch similarity (LPIPS) [29], and the one without reference objects was natural image quality evaluator (NIQE) [30]. Among these, a higher PSNR and SSIM mean better resolution, and lower LPIPS and NIQE mean better resolution.

### 4.2. Training Details

All experiments were performed at 4× scaling factor and NVIDIA GeForce RTX 2080Ti GPUs were used. In order to make a fair performance comparison between our proposed Gram-GAN and the baseline model BebyGAN [17], we referred to the basic experimental configuration of Beby-GAN. Specifically, the optimizer was Adam with parameters $\beta_{1}=0.9$ and $\beta_{2}=0.999$. The size of the input image for the training set was 48×48, and the data was enhanced by random rotation and flip. The size of the mini-batch was set to 8. In Section 3.1, the size of each candidate patch was 4×4 and the magnitude of random affine transform was 0.003. In Section 3.2, we used the features under the 5-th and 11-th convolutional layers to obtain the discriminator perceptual loss. The total number of iterations was 600 k, and every 200 k iterations was a period. The initial learning rate for each period was 1 × 10 ^−4^ and accompanied with the warm-up and cosine decay.

### 4.3. Comparison with State-of-the-Art Technologies

We compare the proposed Gram-GAN with other state-of-the-art perception-driven methods, including SRGAN [3], ESRGAN [11], SFTGAN [13], ESRGAN+ [15] and Beby-GAN [17]. In this paper, we evaluate model performance based on both quantitative and qualitative results, and the details are described in the following section.

#### 4.3.1. Quantitative Results

Table 1 shows the score comparison of the proposed method and other perception-driven methods on each evaluation metric. The proposed Gram-GAN had the highest PSNR and SSIM values among all the methods and also had excellent LPIPS values. The Beby-GAN with single-LR–multiple-HR supervision also had high PSNR, SSIM and LPIPS values. However, its NIQE values were much worse than those of Gram-GAN, which indicated that the visual quality of images generated by Beby-GAN was much lower than Gram-GAN. ESRGAN had low NIQE values, but its PSNR, SSIM and LPIPS values were poor, indicating that the model focused on optimizing the visual quality of the predicted images at the expense of their facticity. The PSNR values reported by SFTGAN were relatively high, whereas its SSIM and LPIPS values were significantly worse than those of Gram-GAN. In conclusion, Gram-GAN showed better improvements in the disadvantage of perception-driven methods generally lacking facticity, and also retained the advantage of the high visual quality of perception-driven methods.

#### 4.3.2. Qualitative Results

**Figure 4:**
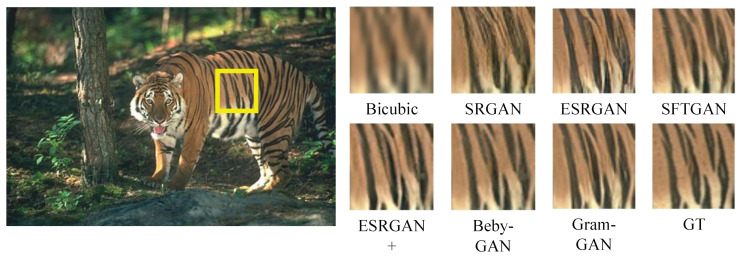
Visual evaluation of state-of-the-art perception-driven methods. Image “108005” from BSD100.

**Figure 5:**
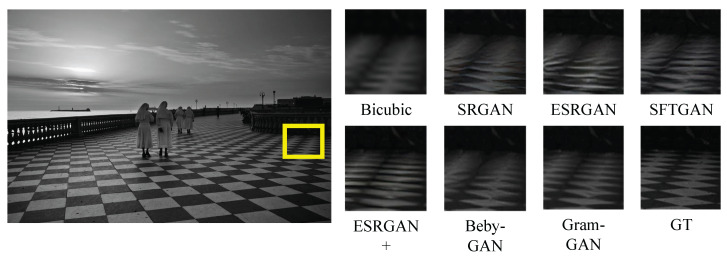
Visual evaluation of state-of-the-art perception-driven methods. Image “img_028” from Urban100.

**Figure 6:**
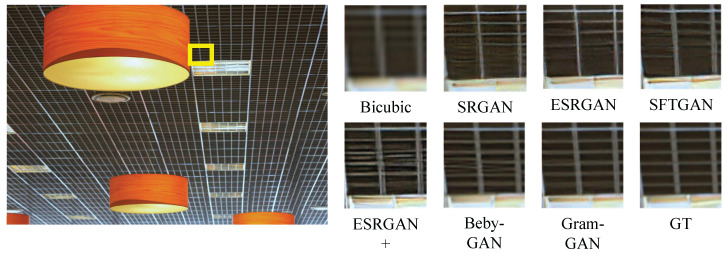
Visual evaluation of state-of-the-art perception-driven methods. Image “img_044” from Urban100.

**Figure 7:**
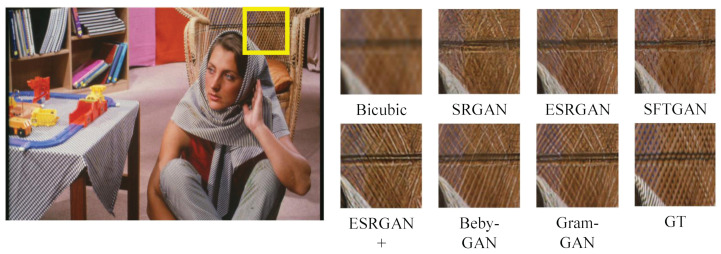
Visual evaluation of state-of-the-art perception-driven methods. Image “barbara” from Set14.

Figure 4, Figure 5, Figure 6 and Figure 7 show the comparison of Gram-GAN and other perception-driven methods in terms of visual effects. Gram-GAN was able to reconstruct texture details closer to GT than other methods. Specifically, Figure 4 highlights the pattern on the tiger. Gram-GAN generates the closest pattern to GT, whereas the other methods either have poor effects in pattern shape or have too many non-realistic artifacts. Figure 5 highlights the ground pattern in the distance. It can be found that the textures generated by all the methods except for Gram-GAN and Beby-GAN were distorted to some extent, and the advantage of Gram-GAN over Beby-GAN was that Gram-GAN could make the pattern in the backward position clear. Figure 6 highlights the lines on the ceiling, and all the methods except for Gram-GAN generated some pseudo lines. Figure 7 highlights the cross-stripes on the chair. Only the proposed Gram-GAN was able to generate dense and clear cross-stripes; the other methods could not achieve this effect.

### 4.4. Ablation Study

**Figure 8:**
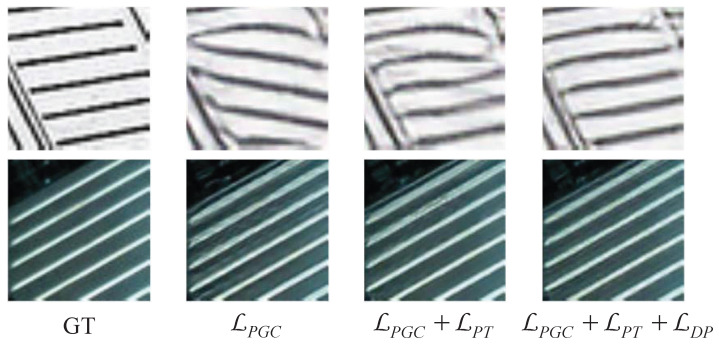
Visual evaluation of the proposed method under the ablation experiment.

In Table 2, we perform the ablation study by superimposing the losses. The initial loss function combination $L_{PGC}$ references to [11], that is, the perceptual loss, content loss and adversarial loss were used. Both of the proposed losses brought some positive effects to the model. PSNR and SSIM values of the model were significantly improved and LPIPS values were reduced after the addition of the patch-wise texture loss. NIQE values of the model were substantially reduced and the values of PSNR and SSIM were further improved after the addition of discriminator perception loss. Figure 8 shows the related visual effects. With the superposition of the proposed loss, the structure of the reconstructed images became gradually close to that of GT images and artifacts arewere eliminated, resulting in higher facticity and visual quality.

## 5. Conclusions

For SR tasks with high visual quality requirements, we first constructed a novel supervision based on the Gram matrix to enhance the flexibility of model inference. Then, a discriminator perceptual loss specifically for SR tasks was proposed to enrich the feature types required for network inference. Finally, a large number of quantitative and qualitative experiments were conducted to verify the effectiveness of the proposed methods, and the necessity of each proposed loss was demonstrated through ablation studies. In future work, considering the high complexity of RRDB, we will focus on optimizing the computational complexity of networks and try to build a high-performance lightweight network.

## Figures and Tables

**Table 1:** Quantitative evaluation of state-of-the-art perception-driven methods.

DataSet	Metric	Bicubic	SRGAN [3]	ESRGAN [11]	SFTGAN [13]	ESRGAN+ [15]	Beby-GAN [17]	Gram-GAN (Ours)
Set5	PSNR	26.69	26.69	26.50	27.26	25.88	27.82	27.97
	SSIM	0.7736	0.7813	0.7565	0.7765	0.7511	0.8004	0.8021
	LPIPS	0.3644	0.1305	0.1080	0.1028	0.1178	0.0875	0.0867
	NIQE	29.56	24.58	18.75	26.87	19.45	25.40	21.34
Set14	PSNR	26.08	25.88	25.52	26.29	25.01	26.86	26.96
	SSIM	0.7467	0.7480	0.7175	0.7397	0.7159	0.7691	0.7710
	LPIPS	0.3870	0.1421	0.1254	0.1177	0.1362	0.1009	0.1003
	NIQE	25.22	18.60	15.19	16.71	16.09	18.45	17.27
BSD100	PSNR	26.07	24.65	24.95	25.71	24.62	26.13	26.32
	SSIM	0.7177	0.7063	0.6785	0.7065	0.6893	0.7347	0.7376
	LPIPS	0.4454	0.1622	0.1428	0.1357	0.1446	0.1192	0.1202
	NIQE	24.35	19.64	16.27	17.23	17.76	21.05	18.53
Urban100	PSNR	24.73	24.04	24.21	25.04	23.98	25.72	25.89
	SSIM	0.7101	0.7209	0.7045	0.7314	0.7182	0.7652	0.7679
	LPIPS	0.4346	0.1534	0.1354	0.1259	0.1334	0.1066	0.1076
	NIQE	20.63	14.93	12.52	13.12	13.38	15.76	14.28

The best performance is highlighted in red (best) and blue (second best).

**Table 2:** Quantitative evaluation of the proposed method under the ablation experiment.

Metric	$L_{\mathrm{PGC}}$	$L_{\mathrm{PGC}}+L_{\mathrm{PT}}$	$L_{\mathrm{PGC}}+L_{\mathrm{PT}}+L_{\mathrm{DP}}$	Set5	Set14	BSD100	Urban100
PSNR	✓			27.72	26.69	26.06	25.59
	✓	✓		27.96	26.97	26.24	25.79
	✓	✓	✓	27.97	26.96	26.32	25.89
SSIM	✓			0.7967	0.7647	0.7290	0.7593
	✓	✓		0.8016	0.7709	0.7353	0.7654
	✓	✓	✓	0.8021	0.7710	0.7376	0.7679
LPIPS	✓			0.0891	0.1031	0.1215	0.1099
	✓	✓		0.0883	0.1021	0.1205	0.1079
	✓	✓	✓	0.0867	0.1003	0.1202	0.1076
NIQE	✓			22.21	18.34	19.32	14.70
	✓	✓		24.36	20.32	19.17	14.65
	✓	✓	✓	21.34	17.27	18.53	14.28

## Data Availability

Our training DIV2K datasets can be obtained online: https://data.vision.ee.ethz.ch/cvl/DIV2K/ (accessed on 21 November 2022). Set5, Set14, BSD100 and Urban100 can be obtained from: https://arxiv.org/abs/1909.11856/ (accessed on 21 November 2022).
